# Multimodal machine learning models identify chemotherapy drugs with prospective clinical efficacy in dogs with relapsed B-cell lymphoma

**DOI:** 10.3389/fonc.2024.1304144

**Published:** 2024-02-08

**Authors:** A. John Callegari, Josephine Tsang, Stanley Park, Deanna Swartzfager, Sheena Kapoor, Kevin Choy, Sungwon Lim

**Affiliations:** ^1^ ImpriMed Inc., Mountain View, CA, United States; ^2^ Department of Oncology, Blue Pearl Seattle Veterinary Specialist, Kirkland, WA, United States

**Keywords:** chemotherapy, machine learning, personalized & precision medicine (PPM), lymphoma, artificial intelligence - AI, rescue therapy, salvage therapy

## Abstract

Dogs with B-cell lymphoma typically respond well to first-line CHOP-based chemotherapy, but there is no standard of care for relapsed patients. To help veterinary oncologists select effective drugs for dogs with lymphoid malignancies such as B-cell lymphoma, we have developed multimodal machine learning models that integrate data from multiple tumor profiling modalities and predict the likelihood of a positive clinical response for 10 commonly used chemotherapy drugs. Here we report on clinical outcomes that occurred after oncologists received a prediction report generated by our models. Remarkably, we found that dogs that received drugs predicted to be effective by the models experienced better clinical outcomes by every metric we analyzed (overall response rate, complete response rate, duration of complete response, patient survival times) relative to other dogs in the study and relative to historical controls.

## Introduction

Diffuse large B cell lymphoma (DLBCL) is the most commonly occurring lymphoma in both dogs and humans (1, 2). In both species, the tumors are typically highly responsive to first-line combination therapies that include cyclophosphamide, doxorubicin, vincristine, and prednisone (CHOP). There is not yet a standard of care for either dogs or humans when patients relapse after first-line therapy (2, 3). Patients may be reinduced with first-line therapy or treated with one of several different rescue therapies (salvage therapies). Thus, in both humans and dogs there is an unmet need for support in identifying the most effective treatment option in the event of relapse.

To help veterinary oncologists rapidly identify the most effective treatments for dogs with lymphoid malignancies like DLBCL, we developed machine learning (ML) models that predict clinical outcomes for 10 different chemotherapy drugs commonly used to treat these malignancies (3). The models predict outcomes derived from medical records by integrating information from two tumor profiling technologies known to yield actionable information with a high frequency: multicolor flow cytometry (4, 5) and ex vivo drug sensitivity testing (3, 6–8). Flow cytometry provides quantitative information about immune cell composition, cell size, and cell granularity at the single-cell level, while ex vivo drug sensitivity testing directly quantifies the cytotoxic effects of different drugs using live tumor cells. ML models like ours, which integrate data from multiple tumor profiling modalities, are termed “multimodal” ML models. Because these models have the potential to increase the accuracy of ML-based precision oncology tools and the frequency with which these tools provide actionable clinical guidance, the development of multimodal ML models is a highly active area of research (9–11).To our knowledge, the study presented here is the first to report on prospective clinical outcomes for cancer patients treated with the assistance of a multimodal ML tool (10).

## Results

We used ML models to generate a prediction report that was provided to oncologists at multiple sites in the US beginning in June of 2020. The report was sent 7 days after live tumor biopsies were received for profiling at our testing facility. In the report, tumor response predictions were presented for each drug on a scale of 0 to 1, with 1 representing the highest likelihood of a positive clinical response (partial response or complete response). We found that there was an approximate correspondence between a prediction score of 0.5 and a 50% probability of a positive response (3). The report provided written guidance on how to interpret the predictions but did not specify how the information should be used to modify treatment plans. Thus, clinicians were free to combine their clinical expertise with the additional information in the prediction report.

The reports were provided to veterinary oncologists at multiple clinics in the US and treatment outcomes were then collected and analyzed. Our primary endpoint for analysis of patient outcomes was patient survival time, but for this study we also analyzed duration of complete response, complete response rate, and overall response rate. Because of the high prevalence of B-cell lymphoma and the short duration of response to therapy in relapsed patients with this cancer type [106 days (12)], patients with relapsed B-cell lymphoma were among the first patients in our population for whom we were able to accumulate a statistically relevant number of prospective survival outcomes. For the current study, we analyzed a cohort of 60 dogs that had relapsed from a prior therapy or therapies at the time that our prediction report was provided (**Supplementary Figure 1**).

Performance of the prediction report was quantified using a matching score analysis commonly employed in human clinical trials where patients are stratified by the degree of matching between recommended and administered drug treatments (13–18). For each dog, the degree to which treatments matched the prediction report was summarized using a matching score similar to those described previously (13–18). The matching score was calculated as the percentage of all administered drug treatments assigned a prediction score greater than 0.5 in our prediction report. We found that the matching scores for this cohort were generally very high, with a median value of 87.5% (**Supplementary Figure 2**).

To examine the relationship between matching scores and clinical outcomes, we split the cohort into two groups at the median matching score value and analyzed outcomes in the two groups (17). One group comprised the lower-matching half of the population while the other group comprised the higher-matching half of the population. A detailed discussion of dichotomization methods is included below in the methods section.

**Figure 1:**
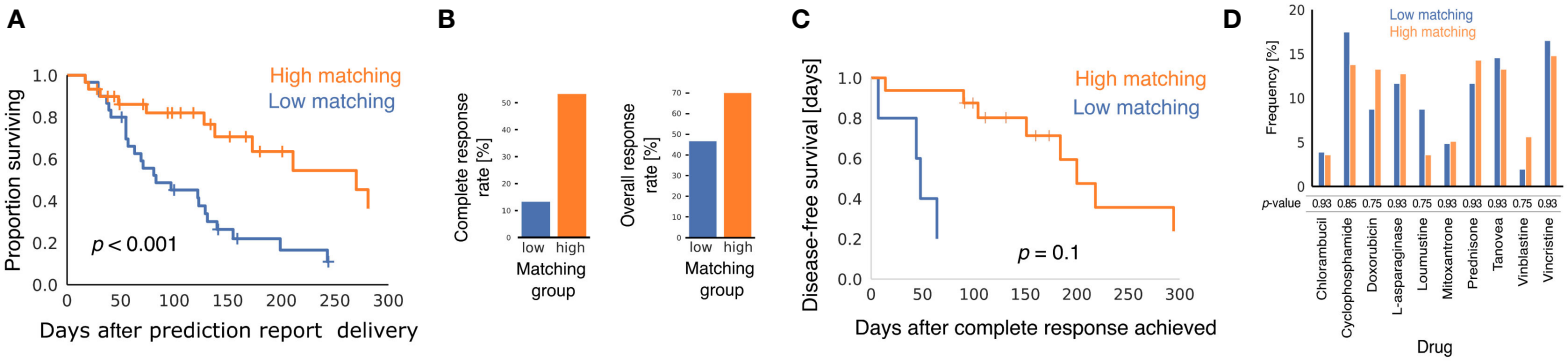
Comparison of clinical outcomes in low matching and high matching groups. **(A)** Kaplan-Meier curves showing survival of dogs after oncologists were provided with multimodal ML predictions. All causes of mortality are included. *p*-value was calculated using the logrank test. **(B)** Bar graphs showing CRR and ORR for the two matching score groups. *p*-values from Fisher's exact test were *p* = 0.002 for CRR and *p* = 0.12 for ORR. **(C)** Kaplan-Meier curves showing disease-free survival after CR for the subset of dogs that experienced CR after their oncologists received multimodal ML predictions (n = 5 for low matching and n = 16 for high matching). *p*-value was calculated using the logrank test. **(D)** Bar graph showing the relative frequencies with which different chemotherapy drugs were predicted to elicit a positive response for dogs in the two matching groups. *p*-values are from the two-sample Z-test with correction for multiple hypothesis testing using the Benjamini-Hochberg method.

Baseline patient and tumor characteristics were similar in the two matching groups (**Supplementary Table 1**), but clinical outcomes were better in the high matching group for every metric we analyzed. Using the Kaplan-Meier estimator to analyze the interval between receipt of the prediction report and death of the patient (**Figure 1A**), we found that patients in the high matching group experienced significantly longer survival times (*p* < 0.001 for the logrank test), with a median survival time of 270 days in the high matching group and 83 days in the low matching group.

Patients in the high matching group experienced both a higher CR rate (CRR) and a higher overall response rate (ORR) than patients in the low matching group (**Figure 1B**) (CRR: 53.3% high, 13.3% low, *p* = 0.002; ORR: 70.0% high, 46.6% low, *p* = 0.12). In patients that experienced a CR, Kaplan-Meier analysis indicated that duration of CR was longer in the high matching group than in the low matching group (**Figure 1C**) (*p* = 0.10 for the logrank test). The statistical power of this survival curve comparison is limited because only five patients in the low matching group experienced a CR. The median duration of a CR was 200 days for the high matching group as compared to 48 days for the low matching group. Thus, the longer survival experienced by the high matching group was accompanied by a similarly extended period of good health during which the lymphoma was in complete remission.

To determine if matching scores were influenced by the drugs predicted to be effective in the report, we analyzed the frequency with which the prediction report contained scores above 0.5 for the chemotherapy drugs in the high matching and low matching groups (**Figure 1D**). No statistically significant difference was found between the relative frequency of these predictions in the two groups for any drug. Thus, matching scores in the low matching group cannot be explained by properties of the drugs predicted to be effective for the dogs in that group. This analysis also suggests that the drug sensitivity of the two matching groups was similar at the population level and that any differences in clinical outcomes were likely attributable to personalization of the drug selection process.

To isolate the effect of matching score from other variables that might confound our analysis of patient survival times, we corrected for tumor grade, cancer stage, and cancer substage using multivariate Cox regression. In both univariate and multivariate Cox regression models, matching score group was the best predictor of patient survival with a hazard ratio (HR) of 0.31 (95% CI 0.15-0.65) in a univariate model and HR of 0.28 (95% CI 0.14-0.59) in a multivariate model (**Table 1**). Thus, consistent with the baseline patient characteristics shown in **Supplementary Table 1**, the markedly longer survival seen in the high matching group cannot easily be explained by the presence of more advanced or aggressive disease in the low matching group.

**Table 1:** Cox proportional hazards models of patient survival.

covariate	Univariate				Multivariate		
	coef	HR (95% CI)	P-value	concordance	coef	HR (95% CI)	P-value
grade	-0.11	0.90 (0.27-2.98)	0.861	0.49	0.33	1.4 (0.41-4.7)	0.604
substage	0.63	1.8 (0.85-4.14)	0.121	0.55	0.96	2.6 (1.1-6.1)	0.027
stage	-0.13	0.87 (0.57-1.34)	0.535	0.52	-0.23	0.80 (0.49-1.3)	0.357
matching group	-1.16	0.31 (0.15-0.65)	0.002	0.57	-1.26	0.28 (0.14-0.59)	<0.001

## Discussion

The clinical outcome advantage of the high matching group relative to the low matching group was observed with every metric examined (ORR, CRR, durations of CRs, survival times) and after correcting for potentially confounding variables using multivariate Cox modeling. The clinical outcome advantage was also evident in all four metrics when we compared high matching group outcomes to historical controls (**Table 2**). Both ORR and CRR values observed in the high matching group were higher than historical control values (ORR: 70% this study, 48% controls; CRR: 53% this study, 27% controls) (19). The median duration of CR of 200 days that we observed in the high matching group was longer than a historical control value of 106 days taken from the mean of 15 rescue therapy studies (12). Thus, the patients in our study group that received treatments matching their multimodal ML predictions to a high degree experienced approximately double the frequency of CR and double the duration of CR compared to historical values reported in the literature. Although patient survival is not uniformly reported in the canine rescue therapy literature, we estimated that historical median survival time after relapse to be 110 days (see methods section for details), which is substantially lower than the value of 270 days that we observed in the high matching group.

**Table 2:** Comparison of high matching group outcomes to internal and historical controls.

metric	high matching group	low matching group internal control	historical control^a^
complete response rate [%]	53^*^	13^*^	27
overall response rate [%]	70^**^	47^**^	48
median duration of complete response [days]	200	48	106
median survival after relapse [days]	270	83	~110

p-value comparisons between high and low matching groups calculated using Fisher's exact: *p = 0.002, **p = 0.12. ^a^See main text for information on historical controls.

To compare the clinical performance of our precision oncology platform with results from other platforms, we compiled mortality hazard ratios (HRs) from a sample of prospective matching score studies in the published literature (**Table 3**) (13–18). A low HR means that reduced mortality was observed for patients in the high matching group. The HR that we report here (0.28, 95% CI 0.14-0.59) is comparable to that of the most performant precision oncology platform in the sample of published values (HR 0.24, 95% CI 0.078–0.76). Among the studies shown in **Table 3**, our study is the only to use computer-automated predictions rather than recommendations from human experts.

**Table 3:** Mortality hazard ratios for high matching group patients in a sample of different precision oncology publications.

year	study	HR	95% CI	n patients
2023	Shaya et al. (13)	0.24	0.078–0.76	18
2023	*this study*	0.28	0.14-0.59	60
2019	Sicklick et al. (14)	0.44	0.19-1.1	69
2019	Rodon et al. (15)	0.48	0.28–0.84	69
2022	Louie et al. (16)	0.54	0.28–1.03	80
2019	Rodon et al. (15)	0.56	0.25–1.3	38
2016	Wheler et al. (17)	0.65	0.43–1.0	188
2022	Charo et al. (18)	0.65	0.34 to 1.25	113

The results reported here strongly support the efficacy of combining clinical knowledge with multimodal ML decision support to optimize rescue therapy outcomes for canine patients with relapsed B-cell lymphoma. We are actively researching application of this technology in human oncology and the impact of tumor mutation profiling data on ML model performance.

## Methods

### Study design

Multimodal ML models were initially developed during a preclinical research stage and then provided to veterinary oncologists throughout the US. The preclinical research was reported in a previous study (3) and clinical research is reported here. An open cohort study design was used to assess the performance of clinical decision support provided by multimodal ML models. Enrollment began in June of 2020 and is continuing at the time of this publication. Informed consent was obtained from pet owners using a form that was approved by the clinical review boards and ethical review committees of participating veterinary hospitals. Veterinary oncologists at multiple sites in the US collected live-cell tumor biopsies from dogs with lymphoid malignancies as described below. Tumor samples were profiled at ImpriMed labs, generating inputs for multimodal ML models. ML prediction reports were provided to oncologists in pdf format with an average turnaround time of seven days from receipt of samples in the labs. Chemotherapy was administered by veterinary oncologists according to the standards used at their treatment sites. Medical records were requested 3 months after delivery of the prediction report and then periodically after that to increase the length of the outcome observation interval. The stopping point for this study was chosen when we estimated that sufficient time had elapsed from the beginning of the enrollment period to assess patient survival in a statistically relevant number of patients.

### Tumor biopsy

Fine-needle aspirates (FNAs) from enlarged lymph nodes were collected at oncology clinics and shipped to the ImpriMed testing lab via overnight courier and processed within 24-72 hours of collection. Cells were maintained at a high level of viability during shipping using ImpriMed Transport Media (ImpriMed Inc., Mountain View, CA) that was optimized for this purpose.

### Inclusion criteria

For this study, we included dogs with B-cell lymphoma that had relapsed from prior cytotoxic chemotherapy when their oncologists were provided with ML prediction reports. Relapse status was reported to us by participating oncologists or determined by inspection of medical records. We performed immunophenotyping and clonality testing on all tumor samples internally at our A2LA-accredited testing lab. Patients were included in this study that were determined to have a clonal rearrangement of a B-cell receptor using PARR and to have the following immunophenotype using flow cytometry: (CD21^+^ or CD79a^+^)CD34^-^CD14^-^CD3^-^CD5^-^. Only dogs that were treated with 3 or more anticancer drug administrations after reception of the prediction report were included. This final inclusion criterion was added to improve the accuracy of the matching scores by guaranteeing a minimal sample size for the calculation. Cohort selection statistics are shown in **Supplementary Figure 1**. The patients who met all of these inclusion criteria had prediction reports delivered to oncologists on their behalf between June 26th, 2020 and November 1st, 2022. Biopsy samples and medical records for patients in the study cohort were provided by 31 veterinarians at 29 clinics in 14 states. Of the 31 veterinarians, 29 were board-certified oncologists, 1 was an oncology resident, and 1 was a general practitioner.

### Tumor profiling

The sensitivity of live tumor cells to 13 different drugs was quantified using a high-throughput ex vivo assay as previously described (3). Tumor cells were profiled at the single-cell level using multicolor flow cytometry and a panel of 9 primary antibodies as previously described (3).

### Collection of clinical information

Baseline patient characteristics (**Supplementary Table 1**) were collected at the time of biopsy or soon afterwards from service request forms or a web portal. Tumor grades were determined by individual oncology practices and may refer to cytology or histopathology results. Patient medical charts and electronic health record exports were emailed to us by oncology clinics three months or more after the biopsy date. Medical records were inspected and drug treatments, tumor responses, and death/euthanasia events were manually entered into spreadsheets. Tumor response annotations were classified into four categories progressive disease (PD), stable disease (SD), partial response (PR), or complete response (CR). We found that some clinicians used RECIST (20) to objectively assign response categories while others recorded qualitative clinical assessments. Medical records collected and analyzed in this fashion were used both to create clinical outcome labels for training ML models and to quantify health outcomes occurring after delivery of ML predictions.

### ML model development

Binary drug response labels were generated from medical records as previously described (3). Briefly, drug treatments followed by SD or PD clinical tumor responses were assigned a value of 0 and drug treatments followed PR or CR were assigned a value of 1. ML models were trained to predict the binary drug response labels for a set of commonly used drugs using features from flow cytometry and ex vivo drug sensitivity assays as previously described (3). Models were updated periodically over the course of the study by retraining existing models with additional data (continual ML) and by adding models for drugs that had previously lacked sufficient data for model development. Continuous accrual of additional training samples was a consequence of our open cohort study design and resulted in an increasing number of samples independently and identically drawn from the same population of dogs. The first generation of models was trained to predict clinical outcomes for 7 different chemotherapy drugs using training data from 463 dogs with known clinical outcomes. During this study, the number of individual drug prediction models increased to 10 and the number of training samples increased to 842 dogs. The models in release v1.0 were random forest models generated using the caret (21) and ranger (22) libraries. The models in releases v2.0 and above were generated using the scikit-learn (23), BayesOpt (24), XGBoost (25), and imbalanced-learn (26) libraries and were either random forest models, elastic net logistic regression models, or voting ensembles composed of multiple different ML models. Predictions for the low and high matching groups were evenly distributed in time, resulting in a similar utilization of the different model versions in the two matching groups (**Supplementary Figure 3**).

### Matching score calculation

Matching score was determined by calculating the percentage of the drug treatments received by a dog that corresponded to drugs with a prediction score above 0.5 in the prediction report:


$$matching\;score=100\times \frac{treatments\;with\;prediction\;score>0.5}{total\;number\;of\;treatments}$$

Only treatments occurring after delivery of the prediction report were included in the calculation. For the purposes of this analysis, a treatment was defined as a 1 week course of a drug that was administered more than once per week, or a single administration of a drug that was given weekly or at lower frequency. To illustrate calculation of the matching score, consider a dog that received 6 weeks of prednisone treatments given twice per week, and 2 infusions of rabacfosadine (trade name Tanovea-CA1) separated by a three week interval. If the dog’s prediction scores for prednisone and rabacfosadine were 0.3 and 0.7 respectively, then the dog received 2 rabacfosadine treatments that matched the ML predictions and 6 prednisone treatments that did not match the ML predictions for a total of 8 treatments. Thus, the matching score for this dog would be 100 * 2/8 = 25%.

Our matching score calculation was slightly different than the calculation most frequently found in the precision oncology literature (13–18). We introduced a modification to the calculation to prevent the score from biasing our outcome statistics towards positive clinical outcomes in the high matching group. Matching score is typically calculated by dividing the number of drugs given that match actionable biomarkers by the total number of actionable biomarkers. When we implemented this standard matching score for our study, we discovered that the high matching group experienced better clinical outcomes even when we shuffled the drug recommendations. In retrospect, it is easy to see why the standard matching score calculation introduces a bias towards positive clinical outcomes in the high matching group. Patients who lived longer tended to receive a greater number of different drugs by virtue of the fact that the oncologist had more time for empirical therapy (i.e. to try more drugs). Thus, any matching score that rewards the total number of drugs administered will bias towards healthier patients regardless of the performance of the precision oncology platform. We eliminated this inherent bias by including the total number of drugs administered in the denominator of our calculation.

### Dichotomization by matching score

Several methods were found in the precision oncology literature for choosing the threshold value used to dichotomize the study cohort into low matching and high matching groups. In the studies we examined, four used the arbitrary threshold value of 50% (13, 14, 16, 27), three adjusted the threshold to create the greatest difference in outcomes between the high and low matching groups (14, 15, 18), and one study used the median matching score (17). We chose the median matching score as the threshold for dichotomization of our cohort because this method offers no opportunity for investigator bias introduced by testing multiple hypotheses about the appropriate threshold value. The clinical outcome advantage associated with higher matching scores was not dependent on the method of dichotomization (**Supplementary Figure 4**).

### Analysis of clinical outcomes

Clinical outcomes data were analyzed using custom Python scripts and statistical functions from Python libraries. **Supplementary Table 1** was automatically generated using the TableOne library (28). The lifelines library (29) was used for Kaplan-Meier statistics and logrank testing. The scipy library (30) was used to compute Fisher’s exact test. The statsmodels (31) library was used to calculate the two-sample Z-test and Benjamini-Hochberg corrections.

### Cox proportional hazards modeling

The lifelines library (29) was used for univariate and multivariate Cox regression. Confounding variables were chosen based on prior evidence of prognostic significance. The proportional hazards assumption of time invariance was verified for each variable using the check_assumptions() method of the CoxPHFitter class. Models were fit using default parameters for the CoxhPHFitter class (baseline_estimation_method = ‘breslow’, penalizer = 0.0, strata = None, l1_ratio = 0.0, n_baseline_knots = None, knots = None, breakpoints = None). Confidence intervals and p-values were generated by CoxhPHFitter during model fitting. Concordance for the multivariate model was 0.62. Concordance values for univariate models are shown in **Table 1**.

### Estimation of survival after relapse for historical control

We estimated that the historical median survival time after initiation of rescue therapy is roughly 110 days by subtracting median time to relapse from median overall survival time [the mean values from 14 published studies were used to derive this estimate (19)].

## Data availability statement

The raw data supporting the conclusions of this article will be made available by the authors, without undue reservation.

## Ethics statement

The animal studies were approved by the Institutional Review Board and/or Ethics Committee of the BluePearl Science and SAGE Veterinary Centers (protocol code IMVLSA1223.18). The studies were conducted in accordance with the local legislation and institutional requirements. Written informed consent was obtained from the owners for the participation of their animals in this study.

## Author contributions

AC: Conceptualization, Data curation, Formal analysis, Investigation, Methodology, Software, Visualization, Writing – original draft, Writing – review & editing. JT: Investigation, Methodology, Writing – review & editing. SP: Data curation, Project administration, Writing – review & editing. DS: Data curation, Writing – review & editing. SK: Investigation, Methodology, Writing – review & editing. KC: Conceptualization, Investigation, Methodology, Writing – review & editing. SL: Conceptualization, Data curation, Funding acquisition, Investigation, Methodology, Project administration, Resources, Supervision, Writing – review & editing.
