# Supplementary material for: Multimodal machine learning models identify chemotherapy drugs with prospective clinical efficacy in dogs with relapsed B-cell lymphoma
Source: Front Oncol. 2024 Feb 8;14:1304144. doi: 10.3389/fonc.2024.1304144 (PMC10881812; doi:10.3389/fonc.2024.1304144)
Supplement: Supplementary file 1 [file DataSheet_1.pdf]

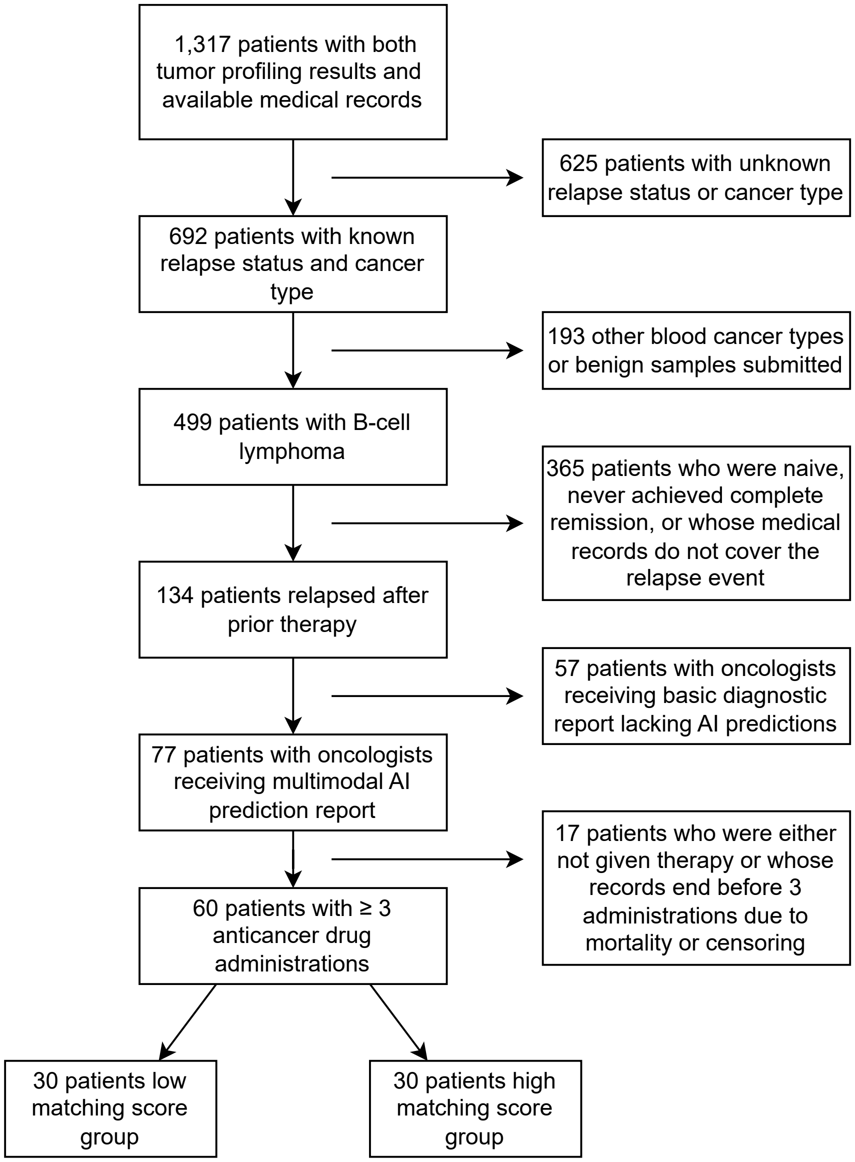

Supplementary Fig. 1 | Cohort selection diagram

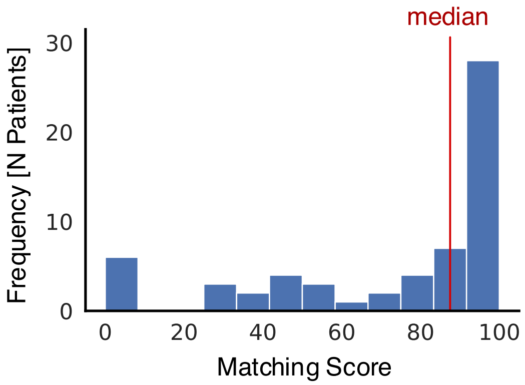

Supplementary Fig. 2 | Frequency of matching scores for relapsed B-cell lymphoma patients. Bar graph showing the frequency of matching score values in the 60 patient cohort. The median matching score value of 87.5% is shown in red. The median was used to dichotomize the population into high matching and low matching groups.

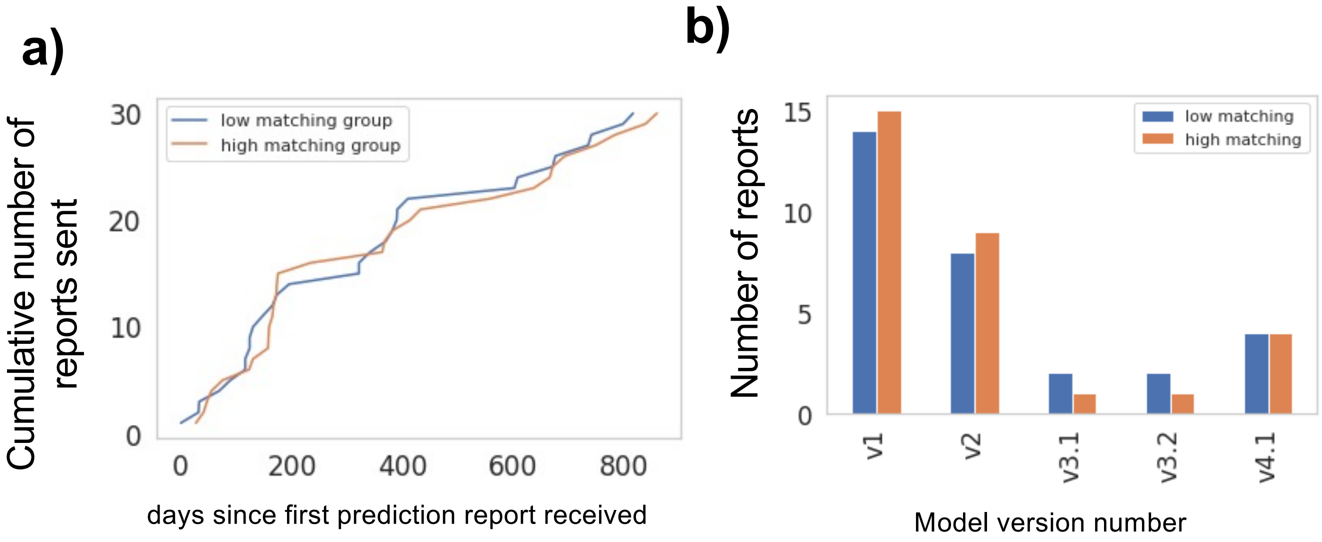

Supplementary Figure 3 | Prediction reports in the low and high matching groups were evenly distributed over time and among ML model versions.

a) Plot showing the cumulative number of reports sent for the two matching groups as a function of the number of days since the beginning of the study. **b)** Plot showing the distribution of model versions used to generate the prediction reports for dogs in the two matching groups.

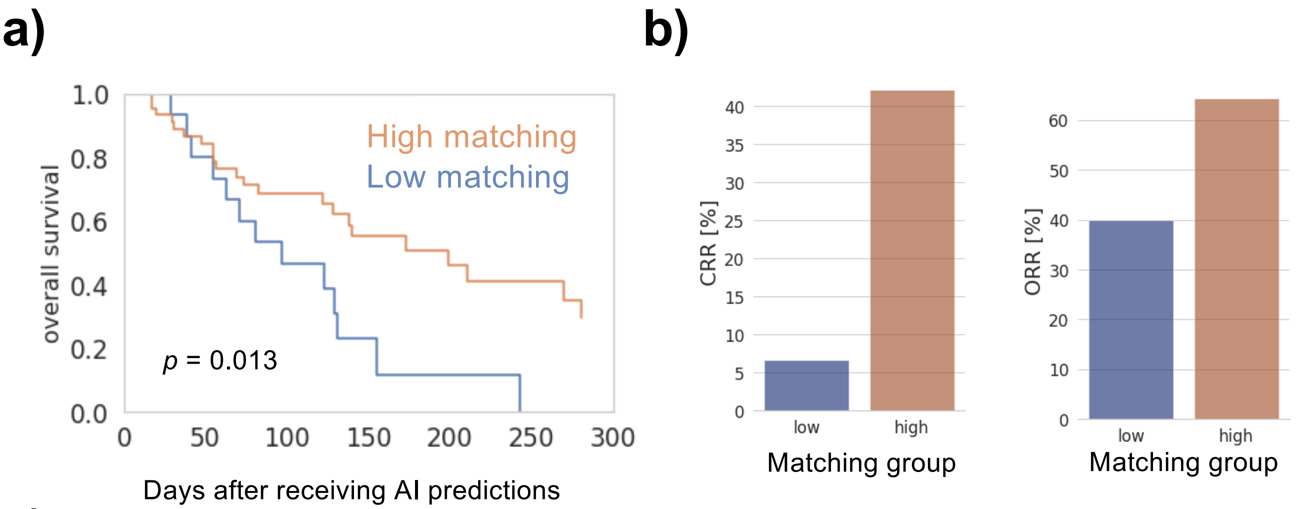

c)

	Univariate			Multivariate	
**covariate**	**coef**	**HR (95% CI)**	**P-value**	**coef**	**HR (95% CI)**
grade	-0.11	0.90 (0.27-2.98)	0.861	0.30	1.4 (0.38-4.8)
substage	0.63	1.8 (0.85-4.14)	0.121	0.67	1.9 (0.85-4.5)
stage	-0.13	0.87 (0.57-1.34)	0.535	-0.23	0.79 (0.51-1.25)
matching group (dichotomization @50%)	-0.871	0.42 (0.21-0.85)	0.016	-0.85	0.43 (0.21-0.89)

Supplementary Figure 4 | Similar clinical outcome advantages were observed in the high matching group relative to the low matching group when patients were dichotomized using a matching score value of 50% instead of the median matching score. With this dichotomization method led, 15 dogs were in the low matching group and 45 dogs were in the high matching group. **a)** Kaplan-Meier analysis of patient survival. **b)** Bar charts showing complete response rates and overall response rates. **c)** Parameters for univariate and multivariate Cox proportional hazards models of patient survival.

Table S1 | Study group characteristics

		Missing	Overall	High matching score group	Low matching score group	*P* value (adjusted)[c]
**number of patients**			60	30	30	
**stage**	ii	9	2 (3.9)	1 (4.0)	1 (3.8)	>0.95
	iii		28 (54.9)	12 (48.0)	16 (61.5)	
	iv		16 (31.4)	8 (32.0)	8 (30.8)	
	v		5 (9.8)	4 (16.0)	1 (3.8)	
**substage**	a	27	26 (78.8)	11 (68.8)	15 (88.2)	>0.95
	b		7 (21.2)	5 (31.2)	2 (11.8)	
**grade**	high	19	36 (87.8)	19 (95.0)	17 (81.0)	>0.95
	intermediate		5 (12.2)	1 (5.0)	4 (19.0)	
**age in days**		0	3220.5 (1076.1)	3345.4 (1080.1)	3095.7 (1075.6)	>0.95
**sex**	female	1	29 (49.2)	10 (34.5)	19 (63.3)	0.909
	male		30 (50.8)	19 (65.5)	11 (36.7)	
**weight in kg**		1	26.4 (13.3)	26.3 (15.2)	26.6 (11.5)	>0.95
**breed**	Australian shepherd	0	3 (5.0)	1 (3.3)	2 (6.7)	>0.95
	German shepherd		5 (8.3)	2 (6.7)	3 (10.0)	
	golden retriever		7 (11.7)	4 (13.3)	3 (10.0)	
	pitbull		7 (11.7)	2 (6.7)	5 (16.7)	
	other		38 (63.3)	21 (70.0)	17 (56.7)	
**day of AI prediction relative to trial start**		0	342.2 (262.2)	348.6 (271.3)	335.8 (257.2)	>0.95
**flow cytometry[a]:**						
**mean FSC-HLin [AU] (cell size)**		2	328.6 (25.4)	326.3 (25.4)	330.8 (25.6)	>0.95
**CD3 % (T-cells)**		2	21.7 (14.1)	21.1 (13.8)	22.3 (14.5)	>0.95
**CD4 % (helper T-cells)**		2	13.5 (10.9)	13.4 (10.6)	13.5 (11.3)	>0.95
**CD5 % (T-cells)**		2	14.0 (13.1)	13.8 (13.7)	14.1 (12.8)	>0.95
**CD8 % (cytotoxic T-cells)**		2	12.1 (11.7)	10.5 (7.1)	13.5 (14.8)	>0.95
**CD14 % (neutrophils)**		2	3.2 (1.6)	3.1 (1.6)	3.3 (1.6)	>0.95
**CD21 % (B-cells)**		2	86.6 (15.2)	84.0 (19.7)	89.0 (9.0)	>0.95
**CD34 % (stem cells)**		2	11.8 (16.3)	13.8 (19.1)	9.9 (13.3)	>0.95
**MHC Class II expression [AU]**		2	391.5 (286.8)	445.2 (350.7)	341.5 (204.4)	>0.95
**clinical data**						
**N drug administrations[d]**		0	9.8 (7.5)	10.3 (9.1)	9.2 (5.6)	>0.95
**complete remission**	not achieved	0	40 (66.7)	14 (46.7)	26 (86.7)	**0.049**
	achieved	1	20 (33.3)	16 (53.3)	4 (13.3)	

All data represent baseline information collected before AI predictions were delivered to oncologists with the exception of the clinical data section at the bottom which refers to treatments and outcomes observed after delivery of AI predictions. For categorical parameters, values refer to number of patients. For numerical parameters, values refer to mean value in group. Numbers in parentheses are percentages for categorical parameters or standard deviations for numerical parameters. [a]Flow cytometric measurements are for the lymphocyte population of tumor fine needle aspirates. [b]MHC expression is presented for the CD21 positive lymphocyte population. [c]*P* values for categorical parameters were estimated using the Chi-squared test. *P* values for numerical parameters were estimated using the T-test. All *P* values were corrected for multiple hypotheses using the Benjamini-Hochberg method. [d]For drugs administered more than once per week, a one week course of treatment was condidered a single administration for the purpose of this analysis.
